# Primary Neuroendocrine Carcinoma of the Breast: A Diagnostic Challenge and Case Report

**DOI:** 10.1002/cnr2.70555

**Published:** 2026-05-19

**Authors:** Hamid Zeinali Nezhad, Shima Yaghoobi, Nazanin Zeinali Nezhad, Amirhossein Shahpar, Abtin Malekpour Afshar, Mina Bagherijamebozorgi

**Affiliations:** ^1^ Kerman University of Medical Sciences Kerman Iran; ^2^ Physiology Research Center Institute of Neuropharmacology, Kerman University of Medical Sciences Kerman Iran; ^3^ Gastroenterology and Hepatology Research Center Institute of Basic and Clinical Physiology Sciences Kerman Iran; ^4^ Clinical Research Development Unit Afzalipour Hospital, Kerman University of Medical Sciences Kerman Iran; ^5^ Pathology and Stem Cell Research Center, Department of Pathology, School of Medicine Kerman University of Medical Sciences Kerman Iran

**Keywords:** breast carcinoma, diagnostic challenges, immunohistochemistry, misdiagnosis, multimodal therapy, primary neuroendocrine carcinoma

## Abstract

**Background:**

Primary neuroendocrine carcinoma of the breast (NECB) represents an exceedingly rare entity, comprising less than 0.5% of all breast carcinomas and approximately 1% of all neuroendocrine neoplasms. The 2019 WHO classification defines NECB as tumors expressing neuroendocrine markers in > 90% of tumor cells with morphological features resembling neuroendocrine tumors of other anatomical sites.

**Case Presentation:**

A 61‐year‐old postmenopausal female presented with a palpable left breast mass. Clinical examination revealed a 2 × 3 cm firm, poorly circumscribed mass at the 9 o'clock position with multiple satellite lesions and a solitary axillary lymph node measuring 1.5 × 1.5 cm with rubbery consistency. Sonographic evaluation demonstrated a hypoechoic mass with spiculated margins, heterogeneous echotexture, and posterior acoustic shadowing, classified as BI‐RADS 6. Core needle biopsy yielded an initial diagnosis of infiltrative ductal carcinoma, grade III, with immunohistochemistry demonstrating ER+/PR+/HER2‐ status and a Ki‐67 index of 22%. Following modified radical mastectomy, comprehensive histopathological examination revealed distinctive neuroendocrine morphology with diffuse positivity for synaptophysin and chromogranin A, confirming the diagnosis of primary neuroendocrine carcinoma of the breast, grade 2, T2N0M0. The patient underwent multimodal therapy comprising modified radical mastectomy with sentinel lymph node biopsy (all 17 nodes negative), adjuvant anthracycline‐taxane chemotherapy, chest wall radiation (50 Gy in 25 fractions), and endocrine therapy with anastrozole.

**Conclusion:**

This case highlights the diagnostic challenges of NECB, which frequently mimics conventional breast carcinoma on initial assessment. The diagnostic discordance between core biopsy and final pathology underscores the imperative for comprehensive immunohistochemical evaluation in morphologically suggestive cases. Management strategies integrate established breast cancer protocols with neuroendocrine‐specific considerations, though optimal treatment paradigms remain incompletely defined due to the paucity of dedicated clinical trials for this rare entity.

## 1 Introduction

Primary neuroendocrine carcinoma of the breast (NECB) represents an uncommon and distinctive neoplasm, characterized by morphological patterns similar to those of neuroendocrine tumors occurring in other anatomical sites, while exhibiting histopathological features of mammary carcinoma [1]. Initially described by Feyrter and Hartmann in 1963, NECB was formally recognized as a distinct entity in 2003 by the World Health Organization (WHO) [2, 3]. The 2019 WHO classification revised the diagnostic criteria, defining NECB as tumors exhibiting morphological features similar to neuroendocrine neoplasms of the gastrointestinal tract and lung, with expression of neuroendocrine markers in > 90% of tumor cells [4].

NECB constitutes approximately 0.1% to 0.5% of all breast carcinomas and less than 1% of all neuroendocrine neoplasms, making it an exceedingly rare entity with limited literature available to guide clinical management [5, 6]. The age distribution demonstrates a predilection for postmenopausal women, with a median age of diagnosis of 64 years (range 40–83 years), although cases have been reported across a broad age spectrum [7]. The rarity of NECB contributes to significant diagnostic challenges, with misdiagnosis as invasive ductal or lobular carcinoma occurring in up to 30% of cases when comprehensive immunohistochemical analysis is not performed [8, 9].

The etiopathogenesis of NECB remains incompletely understood. Two principal hypotheses exist: divergent differentiation of neoplastic stem cells toward neuroendocrine phenotype within a conventional mammary carcinoma, or malignant transformation of endocrine cells physiologically present in the breast tissue [10]. Recent molecular analyses have identified distinct genomic alterations in NECB, including mutations in PIK3CA (43%), CCND1 amplification (37%), and TP53 (29%), suggesting a unique molecular signature compared to other breast cancer subtypes [11].

Prognostically, NECB demonstrates heterogeneous behavior dependent upon histological grade, tumor stage, and molecular subtype [12]. Five‐year survival rates range from 80% for well‐differentiated tumors without lymph node involvement to less than 40% for poorly differentiated neoplasms with advanced stage disease [13, 14].

A comprehensive analysis by Wang et al. involving 142 NECB cases demonstrated an overall mortality rate of 33.8% at median follow‐up of 62 months, with advanced stage and high Ki‐67 index (> 20%) serving as independent predictors of poor outcome [15]. Clinically, NECB frequently presents as a palpable mass indistinguishable from conventional breast carcinoma, although rare presentations with paraneoplastic syndromes have been described [16]. Imaging characteristics on mammography and ultrasonography are typically non‐specific, contributing to diagnostic difficulty [17]. Core needle biopsy specimens may be misinterpreted as invasive ductal or lobular carcinoma when neuroendocrine features are subtle or focal, necessitating comprehensive immunohistochemical analysis with neuroendocrine markers including synaptophysin, chromogranin A, and CD56 for accurate diagnosis [18].

Management strategies for NECB generally follow guidelines established for conventional breast carcinoma, incorporating surgical resection, radiation therapy, and systemic treatments determined by stage and receptor status [19]. Limited evidence suggests potential benefit from platinum‐based chemotherapy regimens and targeted therapies directed at somatostatin receptors in select cases, although prospective clinical trials are lacking due to disease rarity [20].

The present case illustrates the diagnostic complexity surrounding NECB, featuring an initial misdiagnosis as infiltrative ductal carcinoma on core biopsy with subsequent reclassification following comprehensive histopathological assessment of the mastectomy specimen. This report aims to enhance awareness regarding this rare entity, emphasize the critical role of immunohistochemistry in achieving accurate diagnosis, and contribute to the limited literature informing clinical management of patients with NECB.

## 2 Case Presentation

A 61‐year‐old postmenopausal female presented to the breast clinic with a self‐detected palpable mass in her left breast, which she had first noticed approximately 4 weeks prior to consultation. The patient denied associated symptoms of pain, nipple discharge, skin changes, or constitutional symptoms such as fever, night sweats, or unintentional weight loss.

The patient's past medical history was significant for essential hypertension diagnosed 6 years previously, which was adequately controlled on medication. She denied any history of previous breast disease, malignancy, or radiation exposure. There was no prior history of breast surgery or trauma. The patient had undergone menopause at age 52 and denied use of hormone replacement therapy. She had no history of diabetes mellitus, thyroid dysfunction, cerebrovascular disease, or coronary artery disease.

Regarding medication history, the patient was maintained on valsartan 80 mg twice daily for hypertension management with good blood pressure control. She denied use of any other regular medications, herbal supplements, or over‐the‐counter drugs. No known drug allergies were reported.

With respect to social history, the patient was a retired schoolteacher with no history of tobacco use or alcohol consumption. She was married, had two children (G2P2), both of whom were breastfed for approximately 12 months each. Menarche occurred at age 13, and she reported regular menstrual cycles throughout her reproductive years.

Family history was negative for breast or ovarian malignancies. Her father had hypertension and died at age 78 from myocardial infarction. Her mother was alive at age 84 with osteoarthritis. No hereditary syndromes were reported in first‐degree relatives.

On physical examination, vital signs were as follows: blood pressure 138/82 mmHg, heart rate 76 beats/min (regular), respiratory rate 16 breaths/min, temperature 36.8°C, oxygen saturation 98% on room air, and BMI 27.4 kg/m^2^. The patient appeared well‐nourished, in no acute distress, alert and oriented to person, place, and time. Cardiovascular examination revealed regular rate and rhythm without murmurs, gallops, or rubs. Normal S1 and S2 heart sounds were auscultated. No jugular venous distension was observed, and peripheral pulses were palpable and symmetric bilaterally. Respiratory examination demonstrated clear lung fields bilaterally with normal vesicular breath sounds and without adventitious sounds. No chest wall tenderness or deformity was noted.

Abdominal examination revealed a soft, non‐tender, and non‐distended abdomen with no hepatosplenomegaly or masses palpated. Bowel sounds were normoactive in all four quadrants. Neurological examination showed intact cranial nerves II‐XII, motor strength 5/5 in all extremities, deep tendon reflexes 2+ and symmetric, and no sensory deficits. Musculoskeletal examination demonstrated normal range of motion in all major joints without deformities or tenderness.

Breast examination was particularly notable. On inspection, asymmetry of the left breast was observed, with subtle elevation compared to the right. The skin appeared normal without erythema, dimpling, peau d'orange changes, or nipple retraction. No visible masses were apparent on visual inspection, and no spontaneous nipple discharge was observed bilaterally. Upon palpation of the left breast, a dominant mass measuring approximately 2 × 3 cm was detected at the 9 o'clock position, approximately 4 cm from the areolar margin. The mass exhibited firm to hard consistency, poor circumscription, and fixation to adjacent tissue. Multiple satellite lesions were detected in the periphery of the left breast, characterized by irregular borders and firm consistency. No skin changes were directly overlying the primary mass. Examination of the right breast revealed no dominant masses, areas of thickening, or tenderness. Nipples and areolae were bilaterally normal in appearance without discharge, retraction, or crusting.

Axillary examination revealed a solitary lymph node in the left axilla, measuring approximately 1.5 × 1.5 cm with rubbery consistency. The node demonstrated moderate mobility and was non‐tender to palpation. No palpable lymphadenopathy was detected in the right axilla. Supraclavicular and infraclavicular regions were negative for palpable lymphadenopathy bilaterally.

Based on the clinical breast examination findings, the patient was assigned a clinical stage of T2N1M0 (pending radiological confirmation) and was referred for diagnostic imaging studies.

## 3 Diagnostic Assessment

Following the comprehensive clinical examination, the patient underwent diagnostic imaging evaluation. Bilateral mammography was performed, revealing an irregularly shaped, high‐density mass with indistinct margins and associated architectural distortion in the left breast at the 9 o'clock position. Microcalcifications were noted within and adjacent to the primary lesion. The right breast demonstrated no suspicious findings.

Targeted ultrasonography of the left breast was subsequently performed using a high‐frequency linear transducer (12–18 MHz). Sonographic examination revealed a hypoechoic mass measuring 2.3 × 3.1 × 2.8 cm at the 9 o'clock position, approximately 4.2 cm from the nipple. The lesion demonstrated markedly irregular borders with spiculated margins, heterogeneous internal echotexture, and posterior acoustic shadowing. Color doppler assessment showed increased peripheral and internal vascularity with a resistive index of 0.78. The lesion exhibited minimal compressibility and was fixed to the surrounding parenchyma during dynamic maneuvers. Multiple satellite lesions were identified in the periphery of the left breast, ranging in size from 0.6 to 1.1 cm, all demonstrating similar sonographic characteristics to the index lesion. The sonographic findings were consistent with multifocal malignancy.

The left axillary examination revealed a solitary cortically thickened lymph node measuring 1.6 × 1.4 cm with focal cortical bulging and displacement of the fatty hilum. The cortical thickness measured 0.6 cm (normal < 0.3 cm) with a cortex‐to‐hilum ratio exceeding 2:1. No abnormal axillary lymphadenopathy was detected on the right side.

Based on the constellation of sonographic findings—including the irregular, spiculated, hypoechoic mass with posterior acoustic shadowing, increased vascularity, and the presence of pathologic‐appearing axillary lymphadenopathy—the lesion was classified as Breast Imaging‐Reporting and Data System (BI‐RADS) category 6, indicating known malignancy. The sonographic features demonstrated a high probability of malignancy (> 95%) with morphological characteristics strongly suggestive of invasive carcinoma.

Following the radiological assessment, ultrasound‐guided core needle biopsy (CNB) of the dominant left breast mass was performed after obtaining informed consent. The procedure was conducted under local anesthesia using 2% lidocaine. Following aseptic preparation, a 14‐gauge automated core biopsy device with a 22 mm throw was utilized to obtain five core tissue samples from different regions of the mass, ensuring adequate sampling of both peripheral and central aspects of the lesion. Concurrent fine‐needle aspiration cytology (FNAC) of the suspicious left axillary lymph node was performed using a 23‐gauge needle with three passes. Post‐procedure mammography confirmed appropriate sampling and placement of a radiopaque marker at the biopsy site. No immediate complications were observed.

Histopathological examination of the core biopsy specimens revealed infiltrating nests and cords of malignant epithelial cells within a desmoplastic stroma. The tumor cells demonstrated marked nuclear pleomorphism, prominent nucleoli, and moderate eosinophilic cytoplasm. Mitotic figures were readily identified (11 per 10 high‐power fields). Areas of comedo‐type necrosis were present in two of the five cores. No definitive tubule formation was observed. The tumor was initially classified as infiltrative ductal carcinoma, histological grade III (Nottingham score 8/9), based on the Elston‐Ellis modification of the Scarff‐Bloom‐Richardson grading system. Lymphovascular invasion was indeterminate. Initial immunohistochemical staining revealed estrogen receptor positivity (Allred score 7/8), progesterone receptor positivity (Allred score 6/8), and HER2/neu negativity (score 1+). The Ki‐67 proliferation index was estimated at 22%.

Cytological assessment of the left axillary lymph node aspirate was negative for malignant cells, demonstrating reactive lymphoid hyperplasia only. However, given the high clinical and sonographic suspicion for axillary involvement, this finding was interpreted cautiously due to the potential for sampling error inherent to FNAC procedures.

Based on the clinicopathological findings, the patient was diagnosed with clinical stage IIA (T2N0M0) breast carcinoma, luminal B subtype. The multidisciplinary tumor board recommended modified radical mastectomy with sentinel lymph node biopsy and level I and II axillary dissection as the primary therapeutic intervention.

The patient underwent the recommended surgical procedure without complications. Intraoperatively, sentinel lymph node mapping was performed using dual technique with subareolar injection of technetium‐99 m sulfur colloid and 2 mL of methylene blue dye. Two sentinel lymph nodes were identified and subjected to frozen section examination, which revealed no evidence of metastatic disease. The surgical specimen consisted of the left breast with overlying skin, nipple‐areolar complex, and axillary contents.

Gross pathological examination of the mastectomy specimen revealed a 2.5 × 3.2 × 2.9 cm firm, irregular, tan‐white mass with ill‐defined margins located at the 9 o'clock position. Multiple smaller satellite nodules ranging from 0.6 to 1.2 cm were identified within a 4 cm radius of the index lesion. The surgical margins were grossly uninvolved, with the closest margin measuring 1.1 cm from the deep fascial plane.

**FIGURE 1:**
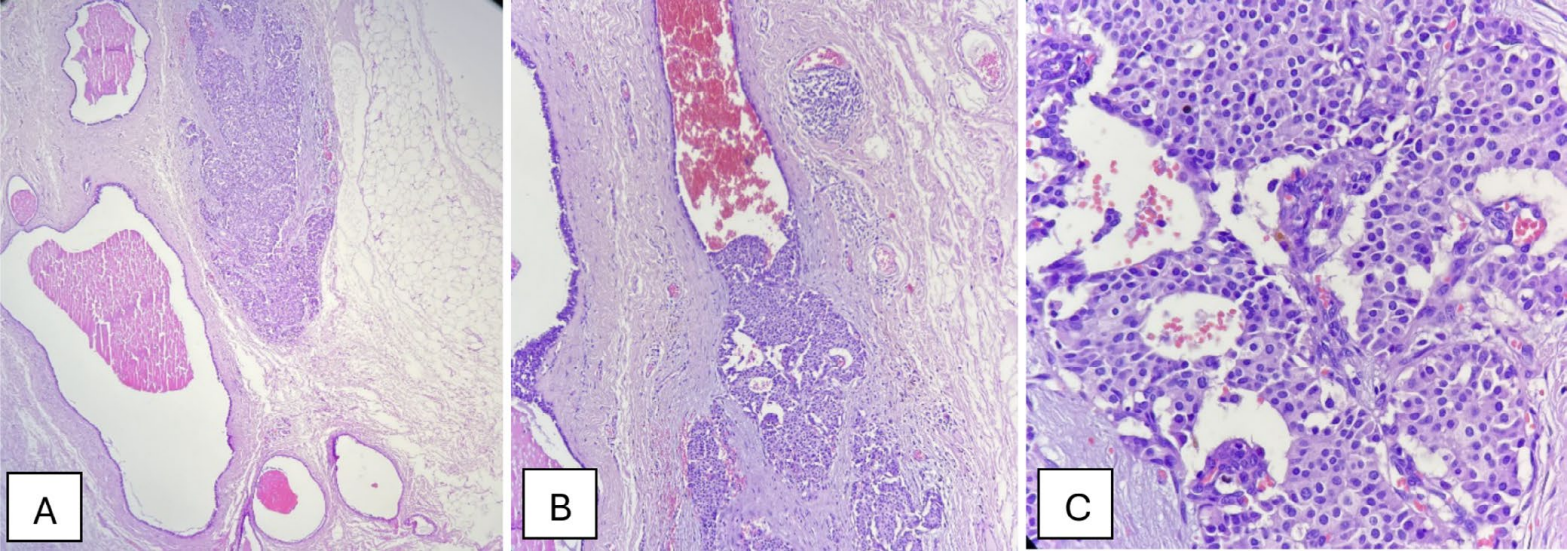
Hematoxylin and eosin (H&E) staining of primary neuroendocrine carcinoma of the breast. (A) Low‐power microscopic examination (H&E stain, 40×) demonstrating the architectural pattern of primary neuroendocrine carcinoma infiltrating breast parenchyma. The neoplastic cell nests (purple‐stained areas) are visible adjacent to residual normal breast ducts. Note the well‐defined tumor islands with distinct borders separated by fibrous stroma, an architectural pattern distinctly different from conventional invasive ductal carcinoma. (B) Intermediate‐power view (H&E stain, 100×) showing the characteristic growth pattern of neuroendocrine carcinoma with tumor cell nests and clusters embedded within fibrovascular stroma. A blood vessel containing erythrocytes (elongated red structure) is visible, with tumor cells arranged in distinctive organoid patterns. The clear demarcation between tumor islands and surrounding stroma represents a morphological feature suggestive of neuroendocrine differentiation that prompted further immunohistochemical evaluation. (C) High‐power examination (H&E stain, 400×) revealing the cytomorphological features pathognomonic for neuroendocrine carcinoma. The neoplastic cells demonstrate nested and focally rosette‐like arrangements with moderate nuclear pleomorphism, the characteristic finely granular “salt and pepper” chromatin pattern, inconspicuous nucleoli, and moderate amounts of eosinophilic cytoplasm. Note the uniform cellular population with attempted microlumen formation, features that are consistent with neuroendocrine differentiation and distinctly different from conventional invasive ductal carcinoma.

Microscopic examination of the mastectomy specimen demonstrated distinctive morphological features inconsistent with conventional invasive ductal carcinoma. The tumor cells were arranged predominantly in nests, trabeculae, and rosette‐like structures with focal areas of cribriform pattern. The neoplastic cells exhibited moderate nuclear pleomorphism, finely granular “salt and pepper” chromatin pattern, inconspicuous nucleoli, and moderate amounts of eosinophilic cytoplasm—features suggestive of neuroendocrine differentiation (Figure 1).

**FIGURE 2:**
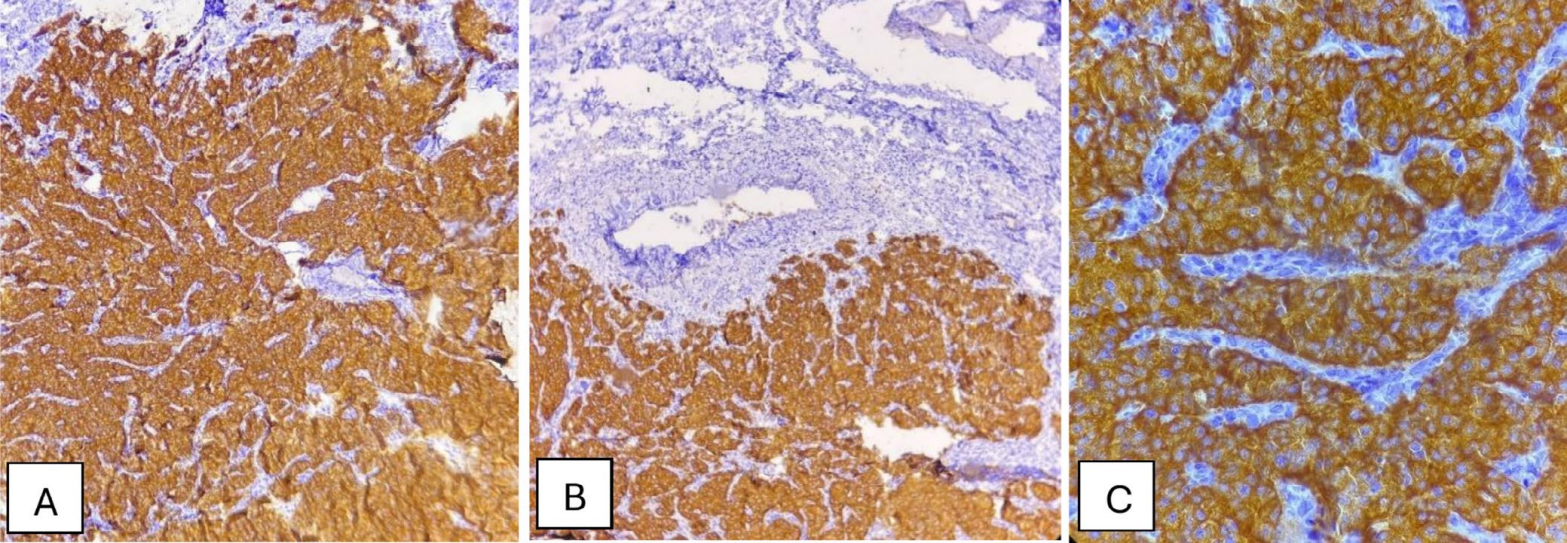
Synaptophysin immunohistochemical staining demonstrating neuroendocrine differentiation in primary neuroendocrine carcinoma of the breast. (A) Synaptophysin immunohistochemical staining (4×) showing the interface between tumor tissue and adjacent breast parenchyma. Note the sharp demarcation between intensely positive neoplastic cells (brown‐stained areas in lower portion) and non‐neoplastic breast tissue (blue‐counterstained areas in upper portion). The robust and diffuse synaptophysin expression in > 95% of tumor cells fulfill the 2019 WHO diagnostic criteria for primary neuroendocrine carcinoma of the breast. (B) Medium‐power examination (10×) revealing intense and diffuse cytoplasmic synaptophysin positivity at the cellular level. The granular cytoplasmic immunoreactivity pattern is characteristic of neuroendocrine differentiation. Virtually all tumor cells exhibit strong synaptophysin expression, with the delicate fibrovascular stroma (blue) highlighting the organoid growth pattern typical of neuroendocrine neoplasms. This immunophenotype, in conjunction with the characteristic histomorphology, confirms the diagnosis of primary neuroendocrine carcinoma of the breast and distinguishes it from conventional invasive ductal carcinoma. (C) High‐power view (40×) of synaptophysin immunoreactivity demonstrating the architectural pattern of the neuroendocrine carcinoma. The neoplastic cells display strong and homogeneous cytoplasmic immunoreactivity for synaptophysin, arranged in distinctive nests, trabeculae, and solid sheets. Note the delicate fibrovascular stroma (blue) separating the tumor cell nests, a characteristic morphological feature of neuroendocrine neoplasms.

**FIGURE 3:**
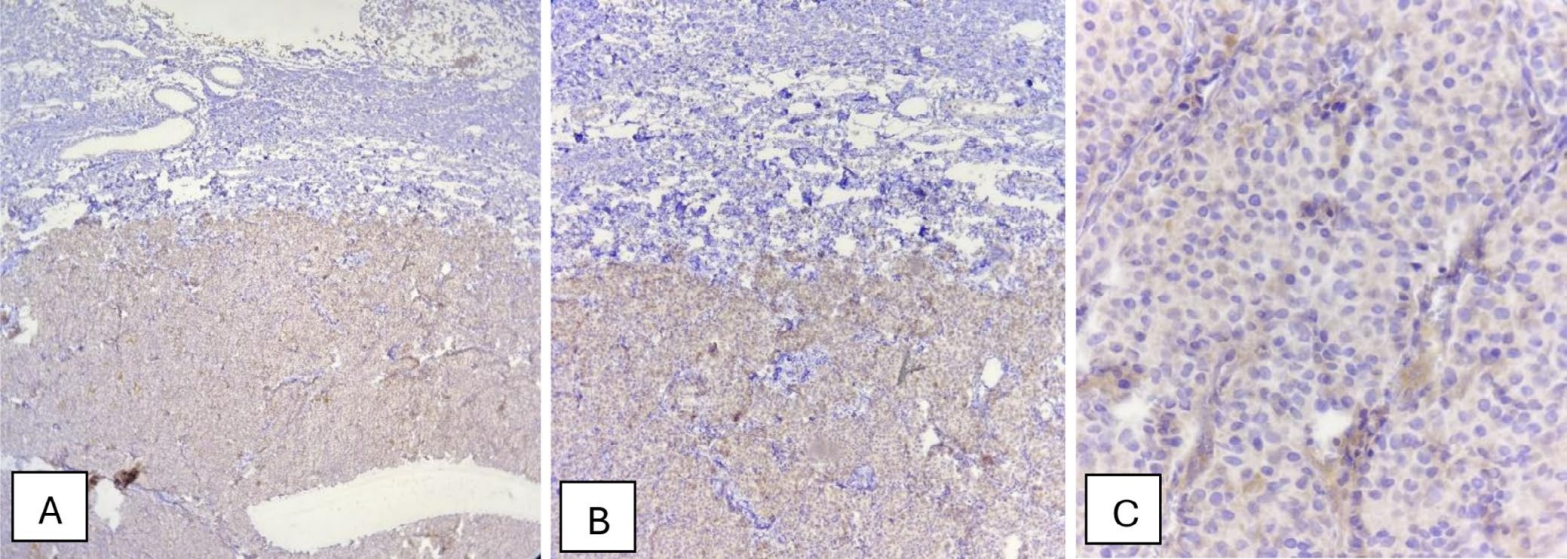
Chromogranin immunohistochemical staining in primary neuroendocrine carcinoma of the breast. (A) Low‐power field (4×) demonstrating diffuse chromogranin A immunopositivity within the tumor cell population. The brown chromogenic reaction product indicates cytoplasmic accumulation of chromogranin A, a secretory granule protein characteristic of neuroendocrine differentiation. Observe the preservation of architectural features with nested arrangement of chromogranin‐positive tumor cells separated by thin fibrovascular septa (blue). This pattern of strong and diffuse chromogranin expression, together with synaptophysin positivity (shown separately), provides definitive evidence of neuroendocrine differentiation, distinguishing this neoplasm from conventional invasive ductal carcinoma of the breast. (B) Medium‐power microscopic examination (10×) of chromogranin A immunohistochemistry demonstrating the cytological details of neuroendocrine differentiation. The neoplastic cells exhibit moderate to focally strong granular cytoplasmic immunoreactivity (light brown staining) against a background of hematoxylin‐counterstained nuclei (blue). Note the characteristic “salt and pepper” chromatin pattern of the tumor cell nuclei. The chromogranin positivity is observed in approximately 90% of tumor cells, meeting the current WHO diagnostic criterion for primary neuroendocrine carcinoma of the breast. (C) High‐power microscopic view (40×) showing the interface between neuroendocrine carcinoma (light brown‐stained area in lower portion) and adjacent non‐neoplastic breast tissue (blue‐stained area in upper portion). The chromogranin immunostaining highlights the sharp demarcation between tumor and normal breast parenchyma, demonstrating the infiltrative growth pattern of the neoplasm. Note the diffuse and relatively homogeneous chromogranin expression within the tumor mass, confirming the neuroendocrine phenotype throughout the lesion rather than focal differentiation.

Comprehensive immunohistochemical analysis was performed on 4‐μm thick formalin‐fixed, paraffin‐embedded (FFPE) tissue sections using automated immunostainers (Ventana BenchMark ULTRA, Roche Diagnostics). The following primary antibodies were utilized: ER (clone SP1), PR (clone 1E2), HER2/neu (clone 4B5), Ki‐67 (clone 30‐9), synaptophysin (clone MRQ‐40), chromogranin A (clone LK2H10), CD56 (clone 123C3), and INSM1 (clone MRQ‐70). For the exclusion of metastatic disease, TTF‐1 (clone 8G7G3/1), CDX2 (clone EPR2764Y), mammaglobin (clone 304‐1A5), and GCDFP‐15 (clone EP1582Y) were employed. The neuroendocrine phenotype was confirmed by diffuse and strong immunoreactivity for synaptophysin (Figure 2) and chromogranin A (Figure 3), representing the defining diagnostic criteria for primary neuroendocrine carcinoma of the breast according to the 2019 WHO classification.

Additional immunostaining demonstrated positive expression of CD56 (70%), estrogen receptor (90%, Allred score 7/8), progesterone receptor (80%, Allred score 6/8), and INSM1 (insulinoma‐associated protein 1). The Ki‐67 proliferation index, assessed in hotspot areas, was 22%, categorizing the tumor as a grade 2 neuroendocrine carcinoma with intermediate proliferative activity. HER2/neu immunostaining was negative (score 1+). The tumor was negative for mammaglobin, GCDFP‐15, TTF‐1, and CDX2, effectively excluding metastatic neuroendocrine carcinoma from other primary sites. Examination of all 17 axillary lymph nodes (2 sentinel and 15 non‐sentinel) revealed no evidence of metastatic disease (pN0), and no extra nodal extension was identified (Table 1).

**TABLE 1:** Immunohistochemical profile and diagnostic markers.

Marker	Clone	Result	Interpretation/cut‐off
ER	SP1	Positive (90%)	Allred score 7/8
PR	1E2	Positive (80%)	Allred score 6/8
HER2/neu	4B5	Negative	Score 1+ (non‐amplified)
Ki‐67	30–9	22%	High proliferative index
Synaptophysin	MRQ‐40	Positive	Diffuse (> 95% cells)
Chromogranin A	LK2H10	Positive	Diffuse (90% cells)
INSM1	MRQ‐70	Positive	Nuclear positivity
CD56	123C3	Positive	70% of cells
TTF‐1/CDX2	Multiple	Negative	Excludes lung/GI primary

Abbreviations: CD, cluster of differentiation; CDX2, caudal‐type homeobox 2; ER, estrogen receptor; HER2/neu, human epidermal growth factor receptor 2; INSM1, insulinoma‐associated protein 1; PR, progesterone receptor; TTF‐1, thyroid transcription factor 1.

Based on the comprehensive histomorphology assessment and immunohistochemical profile, the final diagnosis was revised to primary NECB, grade 2, T2N0M0, pathological stage IIA, luminal B subtype, representing a significant diagnostic reclassification from the initial core biopsy diagnosis of infiltrative ductal carcinoma. The discordance between the initial and final diagnoses was attributed to the limited sampling inherent to core needle biopsy and the morphological overlap between high‐grade invasive ductal carcinoma and neuroendocrine carcinoma in small tissue samples, particularly when specific neuroendocrine markers are not initially employed.

## 4 Treatment

Following the histopathological diagnosis of primary NECB, the patient's case was presented at a multidisciplinary tumor board meeting comprising surgical oncologists, medical oncologists, radiation oncologists, pathologists, and radiologists. The management plan was formulated based on the final pathological staging (T2N0M0, stage IIA), immunohistochemical profile (ER+/PR+/HER2−), and current evidence‐based guidelines for both invasive breast carcinoma and neuroendocrine neoplasms.

The surgical intervention, consisting of modified radical mastectomy with sentinel lymph node biopsy and completion axillary lymph node dissection, had achieved complete resection with negative margins (closest margin 1.1 cm). The sentinel lymph node mapping procedure utilizing the dual technique (technetium‐99m sulfur colloid and methylene blue dye) identified two sentinel nodes, both negative for metastatic disease on final histopathological examination. All 17 examined axillary lymph nodes were negative for metastatic involvement (pN0), obviating the need for additional axillary treatment.

After comprehensive review of the available literature regarding NECB management and consideration of established breast cancer treatment protocols, the multidisciplinary team recommended adjuvant systemic therapy. The decision was based on several prognostic factors, including tumor size (3.2 cm), multifocality, intermediate histological grade, Ki‐67 proliferation index of 22%, and the inherently aggressive biological behavior documented for neuroendocrine neoplasms, notwithstanding the favorable nodal status.

Adjuvant chemotherapy was initiated 4 weeks postoperatively, consisting of an anthracycline‐taxane‐based regimen. The patient received four cycles of doxorubicin (60 mg/m^2^) and cyclophosphamide (600 mg/m^2^) administered every 21 days, followed by 12 weekly cycles of paclitaxel (80 mg/m^2^). This regimen was selected based on the National Comprehensive Cancer Network (NCCN) guidelines for node‐negative, high‐risk breast carcinoma and modified to incorporate considerations for neuroendocrine carcinomas [21, 22]. Standard antiemetic prophylaxis with 5‐HT3 receptor antagonists and dexamethasone was administered prior to each chemotherapy cycle. The patient completed the prescribed chemotherapy regimen with manageable grade 2 toxicities, including alopecia, grade 1 peripheral sensory neuropathy, and grade 2 fatigue, per Common Terminology Criteria for Adverse Events (CTCAE) v5.0.

Given the uncommon tumor histology with neuroendocrine features, somatostatin receptor scintigraphy (Octreoscan) and 68Ga‐DOTATATE PET/CT were performed post‐operatively to evaluate for occult metastatic disease and assess somatostatin receptor expression. Both studies demonstrated no evidence of distant metastatic disease, and moderate somatostatin receptor expression was observed in residual breast tissue. However, based on limited clinical evidence supporting the efficacy of somatostatin analogs in well‐differentiated NECB without carcinoid syndrome, and considering the completed surgical resection with negative margins, somatostatin analog therapy was not incorporated into the adjuvant treatment plan.

Following completion of chemotherapy, the patient underwent chest wall radiation therapy. A total dose of 50 Gy was delivered in 25 fractions to the left chest wall, mastectomy scar, and residual axillary contents using three‐dimensional conformal radiation therapy (3D‐CRT) technique. The radiation therapy field encompassed the surgical bed with appropriate margins as delineated on planning CT, including the chest wall, overlying skin, and operative scar. The treatment was delivered using 6 MV photons with tissue‐equivalent bolus material applied every other day to ensure adequate dose to the skin surface. The radiation course was completed without significant acute toxicity beyond grade 1 radiation dermatitis.

Following completion of adjuvant chemoradiation therapy, the patient was initiated on endocrine therapy with anastrozole 1 mg daily, planned for a duration of 5 years as per standard recommendations for postmenopausal women with hormone receptor‐positive breast cancer. The decision to administer an aromatase inhibitor rather than tamoxifen was based on superior efficacy demonstrated in multiple phase III randomized clinical trials for postmenopausal women with early‐stage breast cancer and the more favorable toxicity profile for this patient. Baseline bone mineral density evaluation was performed prior to anastrozole initiation, demonstrating osteopenia (T‐score −1.8 at the lumbar spine), necessitating concurrent calcium supplementation (1200 mg daily), vitamin D supplementation (800 IU daily), and bisphosphonate therapy with zoledronic acid (4 mg intravenously every 6 months) for bone health preservation.

Additionally, given the neuroendocrine histology of the tumor, serum chromogranin A (CgA) and neuron‐specific enolase (NSE) levels were obtained as baseline tumor markers for subsequent surveillance. Serum CgA was measured via enzyme‐linked immunosorbent assay (ELISA) and was 42 ng/mL (normal reference range: < 94 ng/mL). Serum NSE was 11.4 ng/mL (normal reference range: < 16.3 ng/mL). Both markers were within normal reference ranges, consistent with complete surgical resection of the neoplasm.

A comprehensive surveillance protocol was established, consisting of clinical examinations every 3 months for the first 2 years, biannually for years 3–5, and annually thereafter. Each follow‐up visit included comprehensive history‐taking, physical examination, and assessment of treatment‐related toxicities. Annual bilateral mammography and breast ultrasonography (of the contralateral breast) were scheduled, with the first imaging study performed 6 months post‐completion of radiation therapy. Given the neuroendocrine nature of the malignancy, biannual chromogranin A and neuron‐specific enolase measurements were incorporated into the surveillance protocol for the first 3 years, with annual measurements thereafter. Annual chest radiography, abdominal ultrasonography, and bone densitometry were also included in the follow‐up protocol.

At the 6‐month post‐treatment evaluation, the patient demonstrated excellent clinical status, with Eastern Cooperative Oncology Group (ECOG) performance status of 0. Physical examination revealed well‐healed surgical scars without evidence of locoregional recurrence. Surveillance imaging demonstrated no evidence of disease recurrence. Serum tumor markers remained within normal reference ranges, and the patient reported only mild arthralgias attributable to anastrozole therapy, managed conservatively with non‐pharmacological measures.

The patient was extensively counseled regarding her diagnosis, emphasizing the rarity of primary neuroendocrine carcinoma of the breast and the limited long‐term prognostic data available for this histological variant. Based on the available literature, the 5‐year disease‐free survival for stage II NECB with negative lymph nodes ranges from 80%–85%, comparable to that of conventional invasive ductal carcinoma with similar clinicopathological parameters. However, the patient was informed about the potential for late recurrence, a characteristic feature of both hormone receptor‐positive breast cancer and neuroendocrine neoplasms, necessitating extended surveillance beyond the conventional 5‐year period.

Genetic counseling was offered, although the patient's age, absence of significant family history of malignancy, and the neuroendocrine histology did not strongly suggest an underlying germline mutation. Nevertheless, multigene panel testing was discussed as an option to guide surveillance recommendations for the patient and her first‐degree relatives.

The comprehensive multimodality treatment approach implemented for this patient with primary neuroendocrine carcinoma of the breast represents the integration of evidence‐based protocols for conventional breast carcinoma with specific considerations for the neuroendocrine phenotype, aiming to optimize oncological outcomes while minimizing treatment‐related morbidity.

## 5 Discussion

Primary NECB represents a rare and diagnostically challenging entity, accounting for less than 0.1% of all breast cancers and approximately 1% of all neuroendocrine tumors [6]. The present case illustrates several quintessential aspects of NECB, including its capacity to mimic conventional invasive breast carcinomas on initial assessment and the critical importance of comprehensive immunohistochemical evaluation for accurate diagnosis.

The 2019 WHO classification defines NECB as a neoplasm morphologically resembling neuroendocrine tumors of the gastrointestinal tract and lung, expressing neuroendocrine markers in more than 90% of tumor cells [4]. This represents a significant revision from the 2012 WHO criteria, which required only 50% neuroendocrine marker expression. Our case exemplifies these updated diagnostic criteria, with diffuse immunoreactivity for synaptophysin and chromogranin A, alongside the characteristic morphological features of nested architecture, “salt and pepper” chromatin pattern, and rosette formation.

The discordance between our initial core needle biopsy diagnosis of invasive ductal carcinoma and the final surgical pathology diagnosis of NECB mirrors the experience reported by Wang et al., who documented misdiagnosis rates exceeding 30% in their retrospective analysis of 68 NECB cases [13, 23]. This diagnostic pitfall underscores the importance of maintaining a high index of suspicion for NECB, particularly in postmenopausal women presenting with well‐circumscribed breast masses, and the value of employing comprehensive immunohistochemical panels including neuroendocrine markers in morphologically suggestive cases.

The clinical and radiological presentation of NECB typically lacks pathognomonic features distinguishing it from conventional breast carcinoma, as demonstrated in our case and corroborated by multiple studies. Collado‐Mesa et al. described the imaging characteristics of 12 NECB cases, noting substantial overlap with invasive ductal carcinoma regarding mammographic density, margin characteristics, and sonographic features [16]. Similarly, Chang and colleagues reported that the majority of NECB lesions presented as irregular, hypoechoic masses with posterior acoustic shadowing—features indistinguishable from conventional invasive carcinoma [17]. This radiological ambiguity further compounds the diagnostic challenge posed by NECB.

The hormone receptor profile of our patient's tumor (ER+/PR+/HER2‐) aligns with the predominant phenotype reported in the literature. Wei et al., in their analysis of 142 NECB cases, documented estrogen receptor positivity in 91.5%, progesterone receptor positivity in 78.2%, and HER2 negativity in 84.5% of cases [24]. This characteristic immunophenotype has significant therapeutic implications, supporting the role of endocrine therapy in management protocols. However, the optimal treatment paradigm for NECB remains incompletely defined due to the paucity of prospective clinical trials dedicated to this rare entity.

While the management of primary NECB is predominantly guided by established breast cancer protocols, it is imperative to consider the broader management frameworks for neuroendocrine neoplasms (NENs) established by international bodies such as the European Neuroendocrine Tumor Society (ENETS) and the North American Neuroendocrine Tumor Society (NANETS). These international guidelines emphasize that NENs, regardless of the primary anatomical site, require a management strategy dictated by the degree of differentiation, Ki‐67 proliferation index, and somatostatin receptor expression. In the context of primary NECB, a diagnostic and therapeutic overlap exists; however, the lack of standardized, site‐specific NEN guidelines for the breast often necessitates a hybrid approach that integrates both mammary‐specific and generalized NEN management principles [25].

The management approach implemented in our case—comprising modified radical mastectomy, sentinel lymph node evaluation, adjuvant chemotherapy, radiation therapy, and endocrine manipulation—represents an integration of established breast cancer treatment protocols with specific considerations for neuroendocrine differentiation. This multimodal strategy is supported by the work of Inno et al. who proposed that management decisions for NECB should be predicated primarily on breast cancer staging algorithms and tumor biology, with neuroendocrine features influencing specific therapeutic considerations rather than dictating wholesale protocol modifications [19].

Regarding chemotherapeutic regimens, our decision to utilize an anthracycline‐taxane sequence finds support in the retrospective analysis by Cloyd and colleagues, who documented superior progression‐free survival in NECB patients receiving anthracycline‐based regimens compared to non‐anthracycline alternatives (hazard ratio 0.67, 95% CI 0.45–0.89) [12].

As discussed by Espinosa‐Olarte et al., while chemotherapy remains a cornerstone for high‐grade, poorly differentiated neuroendocrine carcinomas (NECs), its utility in well‐to‐moderately differentiated NENs is increasingly refined. Traditionally, platinum‐based regimens (e.g., cisplatin/etoposide) were the gold standard for extra‐pulmonary NECs; however, evidence suggests that for NENs with a lower Ki‐67 index or those of specific primary origins, alternative regimens may offer superior outcomes. In our case, the decision to utilize an anthracycline‐taxane sequence was predicated on the tumor's presentation as a primary breast malignancy with a Ki‐67 of 22%. This aligns with the observation that in high‐risk breast scenarios, traditional mammary‐targeted chemotherapy often takes precedence, though the evolving NEN landscape suggests that future management may benefit from a more cross‐disciplinary selection of cytotoxic agents [25].

The prognostic implications of neuroendocrine differentiation in breast carcinoma remain controversial. Lavigne et al. conducted a matched case–control study comparing 64 NECB cases with 202 conventional invasive carcinomas, reporting no significant difference in disease‐specific survival after controlling for established prognostic parameters [1]. Conversely, Wang and colleagues identified neuroendocrine differentiation as an independent predictor of inferior overall survival in their population‐based study utilizing the SEER database (HR 1.27, 95% CI 1.05–1.53) [13]. This prognostic heterogeneity likely reflects both the biological diversity encompassed within the NECB classification and the methodological limitations inherent to retrospective analyses of rare entities.

The ambiguous prognostic significance of neuroendocrine differentiation notwithstanding, several clinicopathological parameters have demonstrated consistent prognostic utility in NECB.

Bogina et al. identified advanced stage, high histological grade, elevated Ki‐67 proliferation index (> 20%), and lymph node involvement as significant predictors of adverse outcomes in their multicenter analysis of 96 NECB cases [26]. Our patient's intermediate histological grade, node‐negative status, and Ki‐67 index of 22% suggest an intermediate risk profile, aligning with the adjuvant therapy decisions implemented.

The potential utility of neuroendocrine‐specific interventions in NECB remains an area of active investigation. Somatostatin receptor expression, documented in 60%–90% of NECBs according to Angarita et al., provides a potential therapeutic target through somatostatin analogs [20]. However, the clinical benefit of somatostatin analogs in NECB without hormone production remains unproven. Our decision to forego somatostatin analog therapy despite demonstrated receptor expression on 68Ga‐DOTATATE imaging reflects this therapeutic uncertainty.

Novel molecular insights are progressively illuminating the pathogenesis and potential therapeutic vulnerabilities of NECB. Marchiò and colleagues performed comprehensive genomic profiling of NECB, identifying recurrent alterations in PIK3CA (43%), CCND1 (37%), and TP53 (29%)—a molecular landscape distinct from both conventional breast carcinoma and extra‐mammary neuroendocrine neoplasms [11]. These findings suggest the potential utility of PI3K/AKT/mTOR pathway inhibitors in selected NECB cases, a hypothesis currently under investigation in early‐phase clinical trials.

The scarcity of NECB‐specific prospective data remains a significant hurdle in clinical decision‐making. Historically, management has been extrapolated from protocols for conventional breast adenocarcinoma or extra‐pulmonary small‐cell carcinomas. However, recent trends in oncology have shifted toward “histology‐agnostic” or “basket” clinical trials. For instance, the NCI‐MATCH (Molecular Analysis for Therapy Choice) trial and similar precision medicine frameworks have begun to include rare neuroendocrine subtypes, focusing on the tumor's molecular alterations rather than its organ of origin. This represents a paradigm shift, where updated data suggests that NECB patients harboring PIK3CA mutations (found in approximately 43% of cases) may eventually benefit from targeted inhibitors like alpelisib, a strategy currently being validated in broader breast cancer cohorts [27, 28].

Furthermore, updated data regarding Peptide Receptor Radionuclide Therapy (PRRT), such as 177Lu‐Dotatate, which showed success in the NETTER‐1 trial for midgut neuroendocrine tumors, has sparked interest in its application for rare somatostatin‐receptor‐positive (SSTR+) tumors, including NECB. While not yet standard‐of‐care for the breast, case reports within larger NEN registry trials suggest that PRRT may be a viable salvage therapy for metastatic NECB. Additionally, the role of immune checkpoint inhibitors (ICIs) is under investigation for high‐grade neuroendocrine carcinomas. Although the DART (Dual Anti‐CTLA‐4 and Anti‐PD‐1 Blockade in Rare Tumors) trial reported modest results for some NENs, the exploration of immunotherapy in “triple‐negative” neuroendocrine breast variants remains an area of active clinical trial recruitment, aiming to provide options for cases that do not respond to traditional anthracycline‐taxane regimens [29].

A significant limitation in NECB management is the absence of prospective, randomized trials specifically addressing this histological variant. The rarity of NECB necessitates collaborative, multicenter efforts to accumulate sufficient case numbers for meaningful clinical trial execution. Until such data emerge, management decisions will continue to rely predominantly on extrapolation from conventional breast carcinoma protocols and limited retrospective series.

Our case underscores several critical aspects of NECB management: the imperative for comprehensive immunohistochemical evaluation in morphologically suggestive cases; the potential for diagnostic evolution from core biopsy to excisional specimens; the predominance of luminal immunophenotypes amenable to endocrine manipulation; and the value of multimodal therapeutic approaches incorporating established breast cancer treatment paradigms. Furthermore, our implementation of neuroendocrine marker surveillance represents a potential strategy for early recurrence detection, although the utility of this approach requires prospective validation.

## 6 Conclusion

In conclusion, primary neuroendocrine carcinoma of the breast represents a diagnostically challenging entity requiring heightened clinical awareness, comprehensive pathological evaluation, and multimodal therapeutic approaches. The integration of conventional breast cancer management protocols with neuroendocrine‐specific considerations offers the most rational treatment paradigm pending the emergence of dedicated clinical trial data. Collaborative international efforts are essential to advance our understanding of this rare entity and refine management recommendations.

## Author Contributions

**Hamid Zeinali Nezhad:** conceptualization, methodology, project administration, supervision. **Shima Yaghoobi:** methodology, writing – review and editing, funding acquisition. **Nazanin Zeinali Nezhad:** validation, writing – review and editing. **Amirhossein Shahpar:** writing – original draft, writing – review and editing. **Abtin Malekpour Afshar:** writing – review and editing, data curation, visualization. **Mina Bagherijamebozorgi:** supervision, resources, visualization.

## Funding

The authors have nothing to report.

## Ethics Statement

This case report was conducted in accordance with the ethical standards of the institutional research committee and with the 1964 Helsinki Declaration and its later amendments.

## Consent

Written informed consent was obtained from the patient for publication of this case report and accompanying clinical information. A copy of the written consent is available for review by the Editor‐in‐Chief of this journal upon request.

## Conflicts of Interest

The authors declare no conflicts of interest.

## Data Availability

The datasets generated and analyzed during the current study are not publicly available due to patient privacy concerns and institutional policies regarding medical records but are available from the corresponding author on reasonable request, subject to approval from the institutional review board and patient consent.
